# Co-delivery of Doxorubicin and Curcumin with Polypeptide Nanocarrier for Synergistic Lymphoma Therapy

**DOI:** 10.1038/s41598-020-64828-1

**Published:** 2020-05-12

**Authors:** Wei Guo, Yuanyuan Song, Wantong Song, Yingmin Liu, Zhihe Liu, Dawei Zhang, Zhaohui Tang, Ou Bai

**Affiliations:** 1 Department of Hematology, The First Hospital of Jilin University, Changchun, China; 2 Key Laboratory of Polymer Ecomaterials, Changchun Institute of Applied Chemistry, Chinese Academy of Sciences, Changchun, China; 3 Department of Pathology, The First Hospital of Jilin University, Changchun, China

**Keywords:** Drug delivery, B-cell lymphoma

## Abstract

The traditional chemotherapy, including Adriamycin (Doxorubicin, DOX), is widely used and is part of the first-line chemotherapy of invasive B cell lymphoma. DOX is nonselective cytotoxic drug and has many adverse effects, which limit its clinical application in combination with other anti-cancer drugs. Optimization of the delivery system targeting tumor microenvironment could be a feasible approach that may have significant clinical significance. Further, combination of DOX with other anticancer drugs, such as curcumin, can enhance the synergistic effects, possibly through epigenetic mechanisms. Hence, we evaluated the efficacy and toxicity of novel nanoparticles that enable the co-delivery of DOX and curcumin in the treatment of invasive B cell lymphoma both *in vivo* and vitro. The polymer nano materials [mPEG-b-P(Glu-co-Phe)] was used to co-load DOX and curcumin (CUR): L-DOX + CUR. DOX signal was measured to determine the ability of the drugs entering the cells by flow cytometry, and the different enrichment areas in the cells were directly observed by confocal microscope. The toxicity of LDOX + CUR was tested by CCK-8 assay in different cells, and the synergistic coefficients were calculated. The cell apoptosis and the possible mechanisms of apoptosis pathways regulation by L-DOX + CUR were examined using flow cytometry and Western Blot. The MTD (maximum tolerable dose) test was performed in mice. Tumor-bearing SCID mice (i.e., BJAB cell) were used to evaluate the *in vivo* efficacy of L-DOX + CUR. L-DOX + CUR, was prepared successfully, and the mole ratio of DOX and CUR fixed in 1.0:1.2. (DOX loading rate 9.7%, CUR loading rate 8.1%). L-DOX + CUR exhibited increased intracellular delivery and the main enrichment area of DOX was nucleus. L-DOX + CUR increased cytotoxicity, induced higher rates of apoptosis, and had synergistic effect, especially in BJAB cells (min CI 0.019). It even had epigenetic effect and affected miRNA levels favorably by down-regulating miR-21, miR-199a and up-regulating miR-98 and miR-200c. Additionally, L-DOX + CUR increased MTD in Kunming mice (i.e., 25 mg/kg), compared to DOX (10 mg/kg) and L-DOX (20 mg/kg). In BJAB cell bearing SCID mice, L-DOX + CUR treatment suppressed tumor growth compared to DOX or L-DOX alone, and exhibited less weight loss in mice. We developed new polymer nanoparticles-mPEG-b-P (Glu-co-Phe) co-loaded with DOX and DUR. L-DOX + CUR exhibited synergistic cytotoxic and apoptotic effects on invasive B cell lymphoma. Treatment of L-DOX + CUR potentiated tumor killing in xenografts and reduced toxicity *in vivo*.

## Introduction

Lymphoma is a malignant tumor that occurs in lymph nodes extranodal lymphoid tissues. Lymphoma is mainly classified into non-Hodgkin lymphoma (NHL) and Hodgkin lymphoma (HL). Mature B-cell lymphoma accounts for about 75% of NHL, the most common of which is diffuse large B-cell lymphoma (DLBCL), which accounts for about 40% of NHL, followed by follicular lymphoma (follicular lymphoma (FL), which accounts for approximately 20% of NHL, mantle cell lymphoma (MCL), which accounts for approximately 7% of NHL, and mucosa-associated lymphatic tissue lymphoma (MALT), which accounts for approximately 2.5% of NHL, followed by Burkitt lymphoma (BL), which accounts for about 3% to 5% of NHL and about 40% of children NHL. Lymphoma is one of the most heterogeneous and most complex diseases diagnosed in hematological tumors. Doxorubicin (DOX) is widely used in cancer therapy and is also an important component of the first-line treatment of lymphoma. Although targeted therapies are continuously developed, DOX is still widely used. However, DOX has similar disadvantages as traditional chemotherapeutic drugs, with poor selectivity and many side effects.

The polymer nanocarriers carry small molecule drugs, use passively targeting enhanced permeability and retention (EPR) effects, relatively target tumor tissues, slowly release the encapsulated drugs, delay the blood circulation time, and can reduce the side effects caused by the non-selection of traditional chemotherapy drugs^1–4^. In nanomaterials, the use of NCA (α-amino acid-N-carboxyanhydrides, α-amino acid) ring-opening polymerization is currently the most economical and efficient method^5^. The polyamino acid prepared by the method is biodegradable and metabolized by a normal metabolic pathway, as a result, the monomer-polymerized polyamino acid is not immunogenic and has high safety profile^6^. Polyamino acids are mostly selected as amphiphilic structures, and the hydrophilic part is mostly polyethylene glycol (PEG). This structure can prolong the blood circulation time of nanosystems^7^. Our collaborator at the Changchun Institute of Applied Chemistry of the Chinese Academy of Sciences has developed methoxy polyethylene glycol-b-poly (L-glutamic acid-co-L-phenylalanine) (mPEG-b-P (Glu-co-Phe)) vector. The carrier supports the doxorubicin by electrostatic interaction between the glutamic acid carboxyl group and the doxorubicin amino group on the basis of the extended cycle time of the PEG, and stabilizes the stability in the system by using the hydrophobic action of the phenyl ring on the phenylalanine to prevent disintegration. Our previous studies have confirmed that the material has almost no toxic side effects^8^. The polymer (polyamino acid) nanocarrier can be loaded with doxorubicin (L-DOX) by utilizing the characteristics of pH sensitivity (disintegration in a weak acid environment) between the nano material and the doxorubicin chemical bond. Therefore, we proposed to use this new carrier to load DOX and explore whether it has advantages in the treatment of lymphoma.

In recent years, many studies on curcumin (CUR) have confirmed that CUR inhibits tumor growth and can play a synergistic effect with a variety of chemotherapy drugs. However, this drug is insoluble in water and has poor bioavailability, so the dosage form will need to be improved. We found that the nanocarrier we selected can load CUR via simple physical embedding method, which increases water solubility. We also found that the nanocarrier can form a hydrophobic core after loading DOX to improve the CUR encapsulation efficiency. Doxorubicin (DOX) combined with Curcumin (CUR) is not a new concept for the treatment of malignant tumors. It has been reported in the literature to reduce the cardiotoxicity of the DOX^9,10^. The cardiotoxicity of DOX is mainly due to the damage of the mitochondrial membrane by the drug, resulting in enhanced mitochondrial membrane penetration, excessive release of cytochrome C, and induction of apoptosis^11,12^. The mitochondrial phosphorylation vector (PiC) is an important component of mitochondrial inner membrane phosphorylation, and the higher the level of this vector, the higher the level of apoptosis^13^. It can prevent mitochondrial damage by reducing lipid peroxidation and stabilizing cell membranes, which also protects cardiomyocytes under the action of DOX^9,10^. CUR treatment (12 mg/L) was given for 2 h before DOX (1 μmol/L), compared with direct application of DOX, significantly reduced the level of apoptosis in H9c2 murine cardiomyocytes^10^. It was also observed that the level of phosphorylated corpuscles increased with time in the DOX treatment group, and decreased significantly in the DOX and CUR co-treatment group^10^.

The DOX nano-treatment is to optimize the transmission system to improve the efficacy and reduce the toxicity. In contrast, CUR is limited by its own chemical structure, insoluble in water, difficult to be directly absorbed by tissues. CUR’s effect is also dose-dependent, and *in vitro* experiments have shown that more than 10 μM and long-term effects (12~24 h) are required to induce apoptosis^14^. In the past ten years, in order to enhance the effect of CUR *in vitro* and *in vivo*, a large number of nanomaterials have been developed. These nanomaterials include poly-nanoparticles, liposomes, micelles, nanoemulsions, nanofibers, solid lipid nanoparticles and solid nanodispersions. These nanomaterials were used mainly to improve the bioavailability of CUR. However, new nanomaterials have been developed to focus on the target delivery of CUR^15,16^. For example, biodegradable liposome poly-nanoparticles carrying CUR exhibit high drug loading and *in vivo* targeted delivery^17^. Some researchers have also loaded DOX and CUR to liposomes and examined their efficacy in mouse colon cancer cell line C26. Liposomes of DOX and CUR can effectively prolong blood circulation time and exhibit stable sustained release, resulting in significantly enhanced cell killing^18^.

In the present study, we first demonstrated that the high molecular weight mPEG-b-P (Glu-co-Phe) can co-load doxorubicin and curcumin and this novel nanoformulation has high anti-lymphoma effect and low toxicity. Interestingly, we found that DOX can promote the loading of CUR. Furthermore, co-delivery of DOX and CUR exhibit synergistic effect *in vitro* and *in vivo*. Our results support the application of polymeric nanomaterials to carry traditional chemotherapeutic drugs and provide a theoretical basis for the treatment of lymphoma with synergistic drugs. The developed novel nano-drug delivery system has considerable clinical value.

## Materials and Methods

### Reagents

Doxorubicin hydrochloride (doxorubicin·HCL) was purchased from Beijing Huafeng Lianbo, [molecular formula C27H29NO11 HCl, molecular weight 579.98, orange-red powder, water-soluble, excitation wavelength 475~485 (nm), emission wavelength 575~585 (nm)] and was stored at 4 °C. Curcumin was purchased from Beijing Huafeng Lianbo [molecular formula C21H20O6, molecular weight 368.39, orange-yellow crystalline powder, solubility: soluble in ethanol, propylene glycol, acetone, glacial acetic acid and alkaline solution, soluble in ethanol, soluble with water, insoluble in cold water and grease, excitation wavelength 400~450 (nm), maximum emission wavelength 530 (nm)] and was stored at 4 °C. 3mPEG-bP (Glu-co-Phe) material was provided by Professor Chen Xuesi of the Institute of Applied Chemistry, Chinese Academy of Sciences. The CCK-8 kit was purchased from KGI for cytotoxicity testing. Annexin V-FITC/PI kit was purchased from KGI for apoptotic flow detection. Total protein extraction kit and protein content detection kit BCA (KGPBCA), purchased from KGI, for total cell protein extraction and protein concentration determination. ECL Plus was purchased from GE Healthcare for the Western Blot chemiluminescence reaction. The nitrocellulose membrane was purchased from Invitrogen for the Western Blot antigen-antibody reaction. Protein molecule Marker was purchased from NEB for Western Blot to indicate protein molecular weight. DAPI (4’,6-diamidino-2-phenylindole) was purchased from Sigma for nuclear staining. Fetal bovine serum, 1640 medium powder was purchased from GIBCO for cell culture. Anti-human β-Actin rabbit monoclonal antibody was purchased from Jinshan Jinqiao. HRP-conjugated secondary antibody was purchased from Sigma for Western Blot. Anti-human Bcl-2, c-MYC, Bax, Bid, caspase-3, caspase-9, PARP, and P53 rabbit-derived monoclonal antibodies were purchased from Abcam as a primary antibody for Western Blot.

### Cell lines

Raji cells (ATCC number CCL-86TM), human Burkitt’s lymphoma cells (EBNA positive), were purchased from the Lilac Park ATCC cell bank (Central Plains). Established by Pulsetft RJV in 1963 from Burkitt’s lymphoma of the left maxilla of an 11-year-old black boy, it is the first passage cell of the first human hematopoietic system. BJAB cells, human large B-cell lymphoma cells, were purchased from the clove garden ATCC cell bank (Shanghai Baili Biotechnology Co., Ltd.). BJAB cells are CD3−, CD10+, CD13−, CD19+, CD20+, CD34−, CD37+, CD38+, HLA-DR+, sm/cyIgG−, sm/cyIgM+, sm/cykappa+, sm/cylambda−. Pfeiffer cells (ATCC number CRL-2632TM), human diffuse large B-cell lymphoma cells (EBV negative) were purchased from the ATCC cell bank (Central Plains). Pfeiffer cells are CD10+, CD19+, CD20+, CD38+, CD2−, CD39−. Human Burkitt cells (Raji), human B-cell lymphoma cells (BJAB), and human large B-cell lymphoma cells (Pfeiffer) were cultured in 1640 medium (GIBCO) containing 10% heat-inactivated fetal bovine serum (GIBCO), penicillin (50 U/mL) and streptomycin (50 U/mL). The cells were cultured in a 37 °C, 5% CO_2_ cell culture incubator.

### Preparation and characterization of drug-loaded nanoparticles

To prepare L-DOX, the mPEG-b-P (Glu-co-Phe) material was dissolved in distilled water and the pH was adjusted to 7.0~7.5 with 0.1 M NaOH. 10 mg of DOX·HCl was dissolved in distilled water, and then added dropwise to the above mPEG-b-P (Glu-co-Phe) solution, and stirred for 24 hours in the dark. The sample was then filtered through a 0.45 um filter, and the filtrate was stored lyophilized at −20 °C. The obtained doxorubicin-loaded nanoparticles were referred as L-DOX. To prepare L-CUR, 10 mg CUR and 100 mg mPEG-b-P (Glu-co-Phe) material was dissolved in 5 mL of dimethylformamide (DMF) and added to 10 mL of deionized water with stirring in the dark for 6 h, deionized water for 24 h. Water was changed 3 times to remove excess DMF. The obtained drug-loaded complex was passed through a 0.45 um filter, and the filtrate was lyophilized and stored at −20 °C. The obtained curcumin nanoparticles obtained is referred as L-CUR.

To prepare L-DOX + CUR, 10 mg DOX·HCI, 10 mg CUR, and 100 mg mPEG-b-P (Glu-co-Phe) were dissolved in 5 mL of DMF, added to 10 mL of deionized water with stirring in the dark for 6 h, deionized water for 24 h. Water was changed 3 times to remove excess DMF. The obtained drug-loaded complex was passed through a 0.45 um filter, and the filtrate was lyophilized and stored at -20 °C. The resulting co-loaded nanoparticles are referred as L-DOX + CUR. Drug loading capacity (DLC) refers to the amount of drug loaded per unit weight or unit volume of microspheres. The absorption of doxorubicin at 480 nm was determined by UV spectrophotometer. The absorption of curcumin at 425 nm was determined. The drug loading amount is calculated by the following formula: Drug loading (wt. %) = (mass of drug in nano drug/total mass of nano drug) × 100.

To measure the drug release, lyophilized nanoparticles were dissolved in 10.0 mL of phosphate buffer solution (pH = 7.4, 6.8 and 5.5), and transfer to dialysis bag (MWCO 3500 Da), and immersed in 40.0 mL of the same PBS release medium, placed in a 37 °C constant temperature shaker box to start the release experiment. At a specific time point (0 h, 2 h, 4 h, 6 h, 8 h, 10 h, 12 h, 24 h, 24 h, 36 h, 48 h, 60 h), 4.0 mL of the solution was removed while adding an equal volume of fresh solution. The concentration of DOX and CUR was measured by a fluorescence spectrometer (λex = 480 nm/425 nm). All experiments were performed in parallel 3 times and the results were expressed as mean ± standard deviation.

### Measurement of cytotoxicity

The cytotoxicity was tested by CCK-8 assay. The cells were planted in 96-well plates (1 to 5 × 10^4^ cells + 90 μL of 1640 medium), and 10 μL of each well was added with different concentrations of DOX, CUR, DOX + CUR, L-DOX, L−CUR, L-DOX + CUR, concentration range of DOX was 0.001 to 10 μM for 24 to 48 h. The absorbance of each well at 450 nm was measured using a microplate reader. Cell viability was calculated according to the following formula. Cell viability (%) = (As-Ab)/(Ac-Ab) × 100%. Among them, As refers to experimental wells (cell-containing medium, CCK-8, drugs), Ac refers to control wells (cell-containing medium, CCK-8, no drugs), and Ab refers to blank wells (without cells and drugs). Coordination Factor (CI) was calculated according to the following formula: CI = (D)1/(Df)1 + (D)2/(Df)2. Wherein f is the survival rate, (D)1 (D)2 refers to the concentration at which f is reached in combination, and (Df)1 (Df)2 refers to the concentration at which f acts alone. CI > 1, indicating mutual antagonism, CI = 1, indicating superposition, CI < 1, indicating synergy, wherein 0.8 ≤ CI is low synergy, 0.6 ≤ CI < 0.8 is moderate synergy, 0.4 ≤ CI < 0.6 Highly synergistic, 0.2 ≤ CI < 0.4 is a strong synergistic effect (18).

### Measurement of endocytosis

Endocytosis and intracellular drug release behavior were studied by laser confocal microscopy and flow cytometry. The time points were selected to be commonly used to study DOX intracellular concentrations for 1 h and 3 h. The cells were seeded in a 6-well plate containing coverslips at a density of (1 to 5) × 10^5^ cells/well, containing 2.0 mL of 1640 medium per well, and added wih drugs (final concentration converted to DOX, 5.0 μM) followed by culture for 1 h or 3 h. When the set time point was reached, the medium was removed by centrifugation (800 rpm for 5 min) and washed twice with PBS of pH 7.4 and the cells were uniformly suspended with 1 mL of PBS. In the control group and CUR-free, the nucleus was stained with DAPI (1 μL each, 1 min, starting from the staining step). After staining, the supernatant was discarded by centrifugation with PBS, and uniformly suspended in 50 μL of PBS. 10 μL of cell suspension was taken in each group at a distance of approximately 20 cm from the vertical table top, and was dropped on a slide-proof slide (1% paraformaldehyde treated), followed by covering the slide, glycerin seal, and observing under a laser scanning confocal microscope. When using a confocal microscope, the excitation wavelength of the sample was first set. A 405 nm laser was applied when observing the DAPI/CUR signal, and a 488 nm laser was applied when observing DOX. The corresponding filter was selected to make the target fluorescent signal display different colors, DAPI is blue fluorescent, CUR is yellow fluorescent, and DOX is red fluorescent. The magnification of the objective lens were adjusted to detect cells under a fluorescence microscope.

### Flow cytometry

The cells were planted in a 6-well plate containing coverslips at a density of (1 to 5) × 10^5^ cells/well, containing 2.0 mL of 1640 medium per well, and added drugs (final concentration converted to DOX, 5.0 μM) followed by culture for 1 h or 3 hours. The medium was then discarded, and cells were wash 3 times with PBS, and were dispended in 0.5 mL PBS. The wavelength of the excitation cytometer was 488 nm, and the DOX signal was detected with a filter having a wavelength greater than 560 nm.

### Measurement of apoptosis

AnnexinV-FITC/PI double staining method was used. The cells were seeded in 6-well plates at 5×10^5^ cells/well, and the drugs (DOX, L-DOX, DOX + CUR, L-DOX + CUR) were added. The final concentration of the drug was converted to DOX 0.5 μM. Cells were treated for 24 h or 48. h. After reaching the set time point, the cells were collected by centrifugation at PBS 1000 rpm for 5 min. 200 μL of Binding Buffer was added and the cells were suspended, and then 1.0 μL of AnnexinV-FITC and 1.0 μL of PI were added to mix, and the blank of the drug-free cells was set. The flow cytometer excitation wavelength was measured at 488 nm, the FITC fluorescence was detected with a filter having a wavelength of 515 nm, and the PI fluorescence was detected with a filter having a wavelength greater than 560 nm.

### Quantitative real-time PCR

Quantitative real-time PCR was performed by reverse-transcribing total RNA (2 μg) using the SuperScript First-Strand Synthesis System for RT-PCR (Invitrogen, China). cDNA was diluted 1:10 to quantify the relative content of mRNA by real-time TaqMan PCR miRNAs (probes and primers) were purchased from Life Science Technology (Beijing, China). Results were normalized relative to house-keeping U6 and compared by the CT (ΔΔCT) method.

### Western blot

The cells were seeded in 6-well plates at 5×10^5^ cells/well, and the drugs (DOX, L-DOX, DOX + CUR, L-DOX + CUR) were added. The final concentration of the drug was converted to DOX 0.5 μM, and the cells were treated for 24 h or 48 h. 5~10 × 10^6^ cells were collected, and centrifuged at 800~1000 rpm for 5 min. The supernatant was discarded, and cells were washed twice with pre-cooled PBS. 1 mL of protein extraction reagent was added to 100 μL of compacted cells (1 μL of protease inhibitor per mL of extractant, 1 μL of DTT, 10 μL of PMSF). The maximum speed of the vortex oscillator was used for 1 min, followed by ice bath for 10~15 min. The oscillation was conducted 2~3 times for 30 sec each time. Centrifuge was performed at 14000×g for 15 min at 4 °C, and the supernatant was transferred to a pre-cooled EP tube to obtain total protein. Protein (40 μg) was separated by 10% SDS-PAGE and stained with antibodies, and analyzed using ECL.

### Animals

Male SCID mice (6~8 weeks old) and male Kunming mice (6~8 weeks old) were purchased from the Animal Experimental Center of Jilin University. Animal feeding, use and handling are carried out in strict accordance with procedures and protocols approved by the Animal Management and Use Committee of Jilin University. SCID mice were used to establish a tumor-bearing model of lymphoma cells. A BJAB cell-bearing SCID mouse model was constructed by injecting BJAB cells (5×10^6^) into the right side of the mouse. Kunming mice were used in the MTD test. It is a distant group of mice with a very low incidence of tumors. It is characterized by strong disease resistance and adaptability, and high reproductive rate and survival rate.

### Determination of the maximum tolerated dose

Male Kunming mice were randomly divided into 15 groups, 10 in each group, and DOX or L-DOX or L-DOX + CUR was injected through the tail vein only once. The doses of each group were 5, 10, 15, 20 and 25 mg DOX/kg. The body weight of the mice was weighed daily and observed for animal death. MTD is defined as the dose of drug that does not cause animal death or a 20% reduction in animal weight during the course of the experiment.

### *In vivo* efficacy experiment

When the tumor volume was about 150 to 200 mm^3^, the mice were randomly divided into 7 groups (6 rats each). On the 0, 4th, and 7th days, PBS, DOX (3 mg/kg), CUR (4.14 mg/kg), DOX + CUR (DOX 3 mg/kg, CUR 4.14 mg/kg) were injected through the tail vein and tumor volume were measured with a vernier caliper. Antitumor effects and drug safety were assessed by measuring tumor volume and body weight of the mice. Tumor volume was calculated by the following formula. When any group of mice has a weight loss of more than 30% or death, treatment was stopped and if the body weight can be restored to more than 80% of the basal body weight, treatment can continue. When the tumor is larger than 1500 mm^3^, it is humanely sacrificed. Tumor volume was calculated using the following formula: Tumor volume = (ab2)/2, where *a* and *b* are the longest and shortest diameters of the tumor, respectively.

### Pathology

At the end of the *in vivo* experiment, SCID mice were anesthetized and the thoracic cavity was opened, and the left ventricle was sequentially perfused with PBS and PBS solution containing 4% paraformaldehyde. At the end of the perfusion, the tumor tissues and main organs (heart, liver, spleen, lung, kidney) were taken out, thoroughly washed with PBS, and the tumor tissues were sealed by paraffin embedding technique. Tumor tissues were cut into 5 μm slices and stained with hematoxylin and eosin (HE), respectively. HE-stained sections were photographed using a microscope.

### Immunohistochemistry

The tumor was sliced, and paraffin sections are routinely dewaxed, hydrated, rinsed with running water, and soaked in water for use. 40 ml of pH 9.0 EDTA concentrate was diluted using distilled water to 2000 ml (1:50 dilution). The solution was put into a stainless-steel pot, placed on the induction cooker, and heated to boiling. The slice on the dyeing rack was quickly put into the pot and immediately put into the pot. After boiling again, the power was adjusted to the minimum (insulation state), the lid was capped for 20 minutes. Slices were left in the room temperature for 10 minutes after the heating time was over, and then was cooled down to room temperature. PBS was used to rinse for 3 min and repeated once. Endogenous peroxidase blocking reagent was added to the section (3% hydrogen peroxide), and incubated for 10 minutes at room temperature, followed by PBS washing 3 times (3 min each). PBS was then removed and ready-to-use primary antibody was added to the section, and incubated for 60 min at room temperature, followed by rinsing with PBS for 5 times (2 min each time). PBS was then removed, and secondary antibody was added on the section and incubated for 8~10 min at room temperature, followed by rinsing with PBS for 3 times (2 min each time). PBS was then removed, and polymer-HRP IgG was added to the sections and incubated for 8~10 min at room temperature, followed by rinsing with PBS for 3 times (2 min each time). 1:25 diluted DAB was then used for 8~10 min. Hematoxylin counterstaining was conducted for 1~2 min, followed by rising with water, 1% hydrochloric acid, gradient alcohol dehydration, xylene transparent, and sealing.

### Statistical analysis

SPSS 24.0 software was used. The statistical method involved chi-square test, single-factor ANOVA test, t-test, P < 0.05, which was considered statistically significant. Graphic production application Origin 8.0 software, Western blot gray value analysis application ImageJ2X software.

## Results

### Preparation of and characterization of L-DOX and L-DOX + CUR

**Figure 1:**
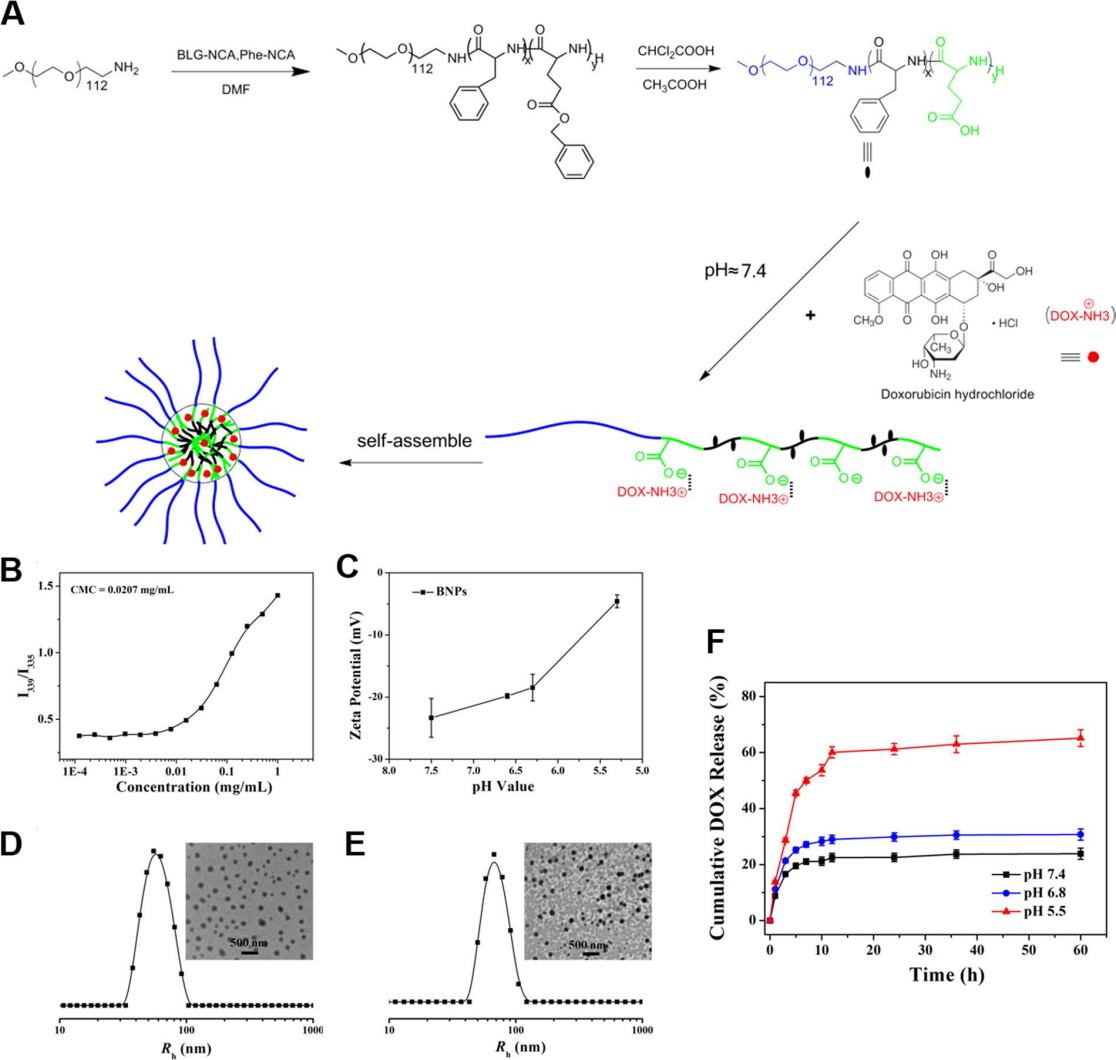
Generations and characterization of nanoparticles. (**A**) Loading DOX onto mPEG-b-P(Glu-co-Phe). (**B**) CMC test; (**C**) blank carrier potential change at different pH; (**D**,**E**) blank carrier and hydrodynamic radius and TEM in aqueous phase after DOX loading. (**F**) in vitro DOX release level of L-DOX in different PBS pH.

mPEG-b-P (Glu-co-Phe) was prepared using α-amino acid - NCA ring-opening polymerization, following exactly previous published protocols^8^. The amphiphilic copolymer can self-assemble into nanoparticles in aqueous solution (Fig. 1A). mPEG-b-P (Glu-co-Phe) exhibits water solubility, and the benzene ring enhances the stability of the nanoparticles by hydrophobic action. The hydrodynamic radius (Rh) of the measured blank nanoparticles and L-DOX were 59.6 ± 13.7 and 70.2 ± 14.2 nm, respectively (Fig. 1B–E). The TEM images showed that the blank nanoparticles and L-DOX exhibited a uniform spherical distribution. The prepared L-DOX drug loading was 10.6%. L-DOX release showed significant pH sensitivity in PBS (Fig. 1F). mPEG-b-P (Glu-co-Phe) exhibits negative electricity. As the pH gradually decreases from 7.4, the potential gradually increases, suggesting that the negative electricity is weakened under weak acid conditions, reducing the electrostatic interaction between the carrier and DOX, and the latter is “extruded”.

To prepare L-CUR, CUR is mixed with mPEG-b-P (Glu-co-Phe) using the principle of physical embedding. However, the encapsulation efficiency is not high, and the drug loading is 1.14%. To prepare L-DOX + CUR, we applied static electricity plus physical embedding. The hydrophobic core formed by DOX can help CUR be better embedded in the core. The drug loading is 9.7%, CUR 8.1%, and DOX: CUR 1: 1.23 (molar ratio). All the combined ratios in this study used this ratio. The results showed that mPEG-b-P (Glu-co-Phe) can successfully encapsulate DOX + CUR and adding DOX can significantly increase the drug loading of CUR and exhibit water solubility. The drug release is similar to 3.1.1.2, and only the absorption wavelength is set differently during CUR detection (data not shown).

### Endocytosis of DOX

**Figure 2:**
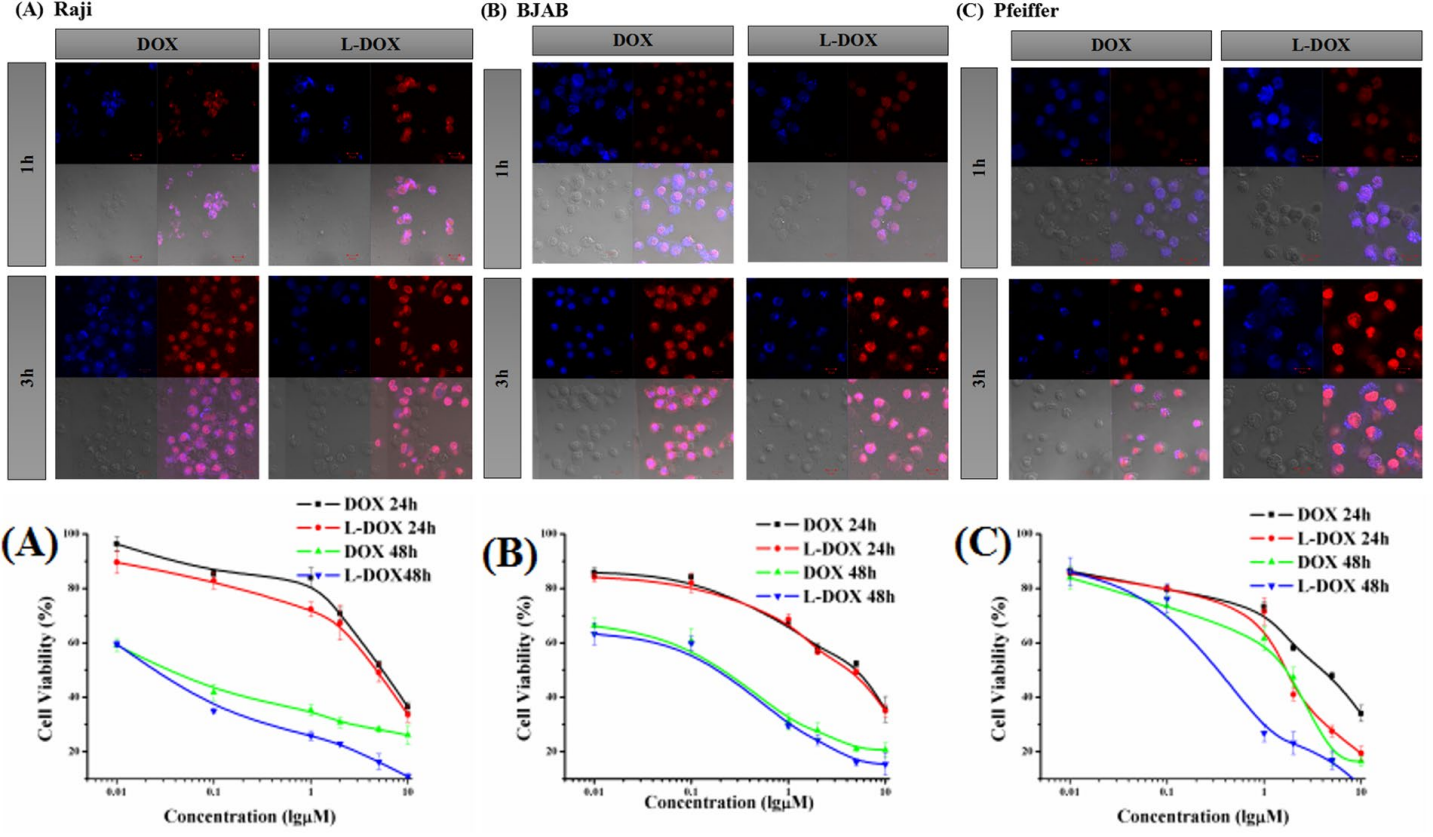
Endocytosis of DOX and cell viability. Upper panels show confocal microscopy results of DOX signal from (**A**) Raji cell (**B**) BJAB cell and (**C**) Pfeiffer cell after incubated by DOX or L-DOX for 1 h or 3 h. (40×). The lower panels show the cell viability of (**A**) Raji cell, (**B**) BJAB cell, and (**C**) Pfeiffer cell in vitro with DOX and L-DOX. CCK-8 assays were performed. Data shown are means ± SD from three separate experiments (n = 3).

We observed the intracellular distribution of the drug at different time points (1 h and 3 h) in DOX group and L-DOX group by laser confocal microscopy. The drug concentration was equivalent to DOX 5.0 μM. The fluorescence parameters of the confocal microscope were adjusted using DOX 3 h as a control. We found that DOX was mainly distributed in the nucleus (Fig. 2), and the signaling intensity was increased after 3 h incubation compared to 1 h. Additionally, L-DOX fluorescence signal was mainly distributed in the nucleus, and the fluorescence intensity was higher than DOX at the same time point.

### Cell viability

**Figure 3:**
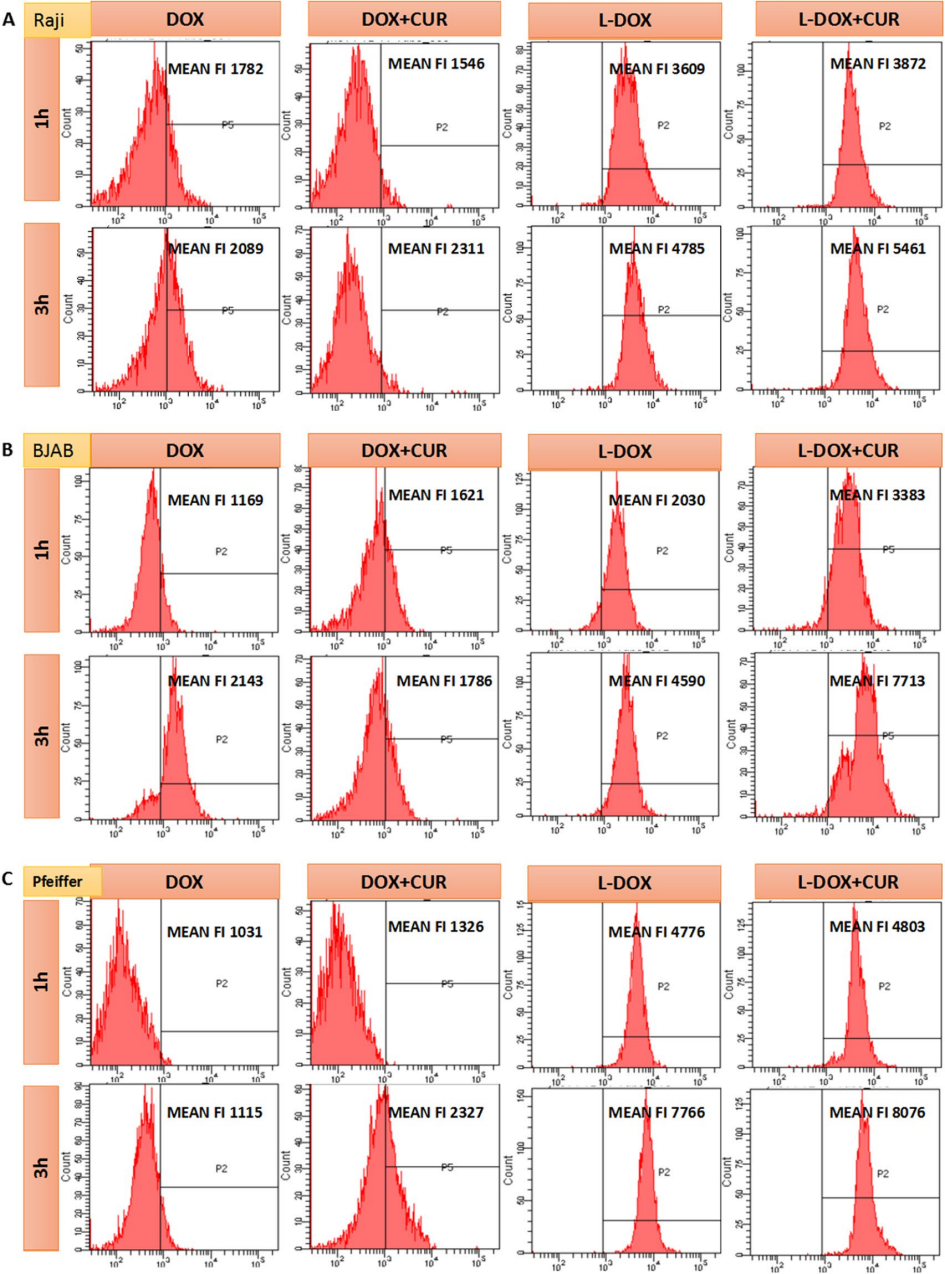
Comparison of DOX concentrations in lymphoma cells. Flow cytometry results of mean DOX fluorescence intensity in different groups on (**A**) Raji cell, (**B**) BJAB cell, and (**C**) Pfeiffer cell. The groups are DOX group, DOX + CUR group, L-DOX group and L-DOX + CUR group. All groups take the final dose equal to 5 μM DOX, and incubation time was 1 h or 3 h.

After treatment with DOX (0.01~10) μM in RaX, BJAB and Pfeiffer cells for 24 h and 48 h, respectively, the cells were observed under an inverted microscope. The degree of inhibition was aggravated, which was manifested by obvious cell membrane shrinkage, chromatin condensation in the nucleus, and partial cell disintegration (not shown). The cell viability results are shown in Fig. 3 and Table 1. The IC50 of 48 h and 24 h was compared. The former was lower than the latter, and the difference was significant, P = 0.000, suggesting the cytotoxicity of DOX and L-DOX was enhanced as the duration was prolonged. Although each cell line showed that the IC50 of the L-DOX group was lower than that of the DOX group, there was difference between cell lines. The IC50 of the Raji cells after 24 h showed that the L-DOX was lower than the DOX group (4.62 μM vs. 5.64 μM, P = 0.046). The IC50 of Pfeiffer cells showed L-DOX was lower than the DOX group (1.69 μM vs. 4.02 μM P = 0.000, 0.34 μM vs. 1.62 μM, P = 0.000) for both 24 h and 48 h. These results proved that L-DOX and DOX showed stronger cytotoxicity over time, which was consistent with the results of confocal microscopy. The toxicity of L-DOX to Raji and Pfeiffer cells was significantly stronger than that of DOX.

**Table 1:** The IC_50_ (μM) of DOX or L-DOX on Raji and BJAB and Pfeiffer cell lines at 24 h and 48 h (±SD).

	Raji		*P*	BJAB		*P*	Pfeiffer		*P*
	DOX	L-DOX		DOX	L-DOX		DOX	L-DOX	
24 h	5.64 ± 0.453	4.62 ± 0.500	0.046	5.04 ± 0.501	4.41 ± 0.400	0.289	4.02 ± 0.352	1.69 ± 0.151	<0.001
48 h	0.04 ± 0.040	0.02 ± 0.002	1.000	0.21 ± 0.025	0.19 ± 0.015	1.000	1.62 ± 0.101	0.34 ± 0.035	<0.001

### Comparison of DOX concentrations in lymphoma cells

The results of flow cytometry are showed in Fig. 3. In Raji, BJAB, Pfeiffer cells, the fluorescence intensity of DOX increased in 3 h compared with 1 h. Furthermore, in the same time point, DOX fluorescence intensity of L-DOX + CUR group was higher than L-DOX group, which was higher than DOX group. The results demonstrated that L-DOX + CUR can be effectively endocytosed by three lymphoma cells, which is stronger than L-DOX. At the same time, we found that although endocytosis was enhanced after L-DOX + CUR in all 3 cell lines, this enhancement was not shown in the DOX + CUR group. For instance, in Raji cells after 1 h treatment of DOX + CUR, and in BJAB cells after 3 h treatment of DOX + CUR, the fluorescence intensity of DOX + CUR was not significantly different from that of DOX. These results showed that the L-DOX + CUR system enhances the ability of the active ingredient DOX to enter cells, and such enhancement is not apparent in the DOX + CUR system.

### Comparison of intracellular DOX and DUR concentration in lymphoma cells

**Figure 4:**
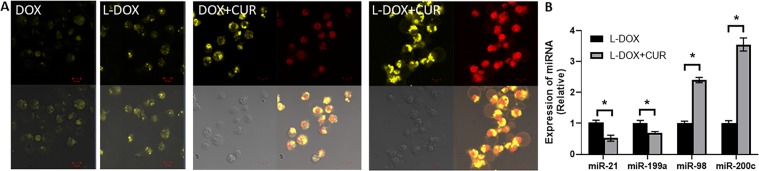
Comparison of intracellular DOX and CUR concentration in lymphoma cells, along with epigenetic affect on miRNAs. (**A**) Confocal microscopy results (40×) of CUR and DOX signal on BJAB cell incubated with different drugs for 3 h. The groups are DOX group, DOX + CUR group, L-DOX group and L-DOX + CUR group. (**B**) miRNA levels were also tested in L-DOX vs LDOX + CUR groups by quantitative RT-PCR. All groups take the final dose equal to 5 μM DOX and 6.15 μM CUR. *p < 0.01.

Because the CUR excitation wavelength is 410~425 nm and the DAPI excitation wavelength is 340 nm, we used the 405 nm laser in the confocal microscope (Zeiss LSM780). Therefore, in order to simultaneously evaluate the CUR and DOX + CUR entering the cell, CUR, L-CUR, DOX + CUR, and L-DOX + CUR were used to treat Raji, BJAB, and Pfeiffer cells respectively. It was found that CUR fluorescence (not shown) was observed after at least 3 hours, and the three cell lines exhibited similar results. Figure 4A shows the results in BJAB cells. It can be seen that the enrichment areas of CUR and DOX are different. DOX has been confirmed to be enriched in the nucleus and CUR is mainly enriched in the extranuclear structure, suggesting that its mechanism of action of CUR is different from that of DOX. The fluorescence signal of L-CUR was slightly stronger than CUR, indicating that the single-agent CUR cannot enter the cell membrane after 3 hours of action, and the ability to enter through endocytosis is enhanced after being loaded in nanoparticles. In the DOX + CUR group, the CUR signal was enhanced compared with the single-agent CUR group. In the L-DOX + CUR group, the CUR signal was further enhanced compared to DOX + CUR group. It suggested that mPEG-b-P (Glu-co-Phe) can enhance the ability of the drug to enter the cell and target release the drug, and CUR may have a synergistic effect with DOX. On further testing of epigenetic effects, it was found that L-DOX + CUR group had a significant inhibitory effect on the oncogenic miRNAs miR-21 and miR-199a (Fig. 4B). At the same time, the tumor suppressor miRNAs were up-regulated, such as miR-98 and miR-200c.

### Comparison of effects of drugs on lymphoma cell proliferation

**Figure 5:**
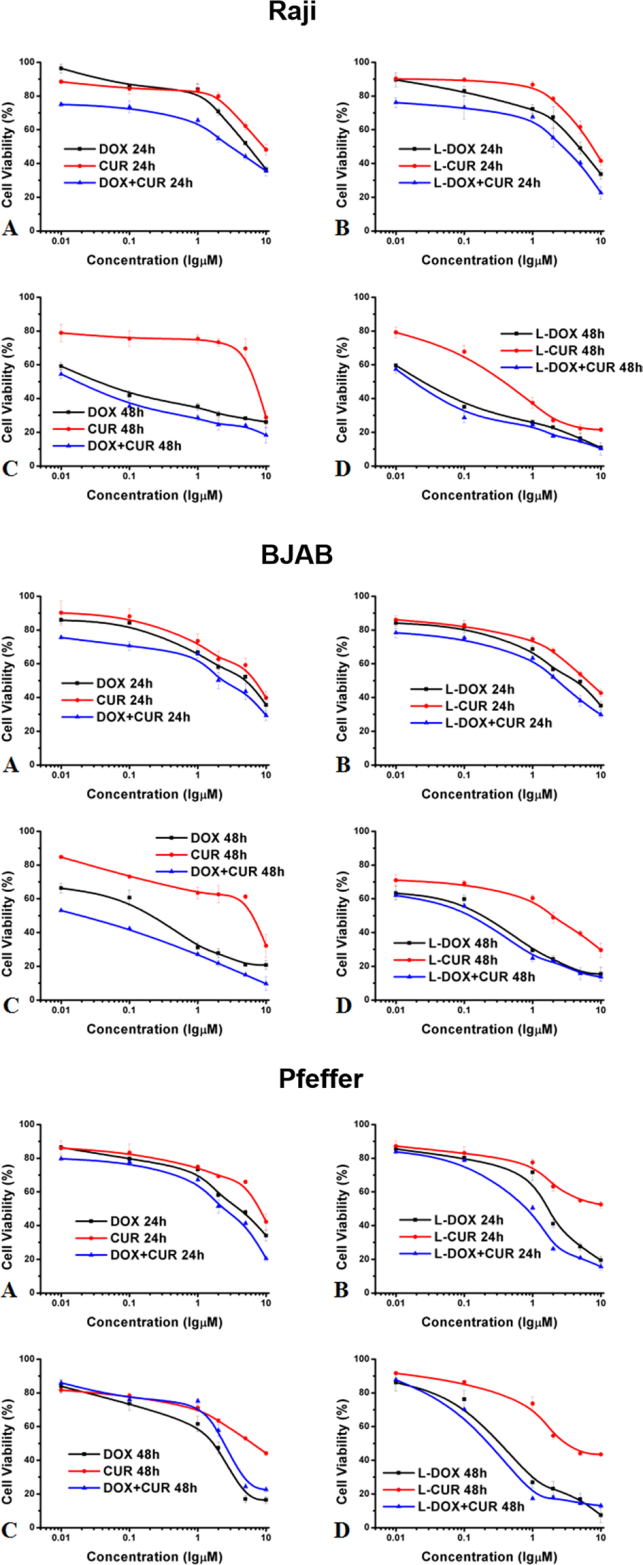
Comparison of effects of drugs on lymphoma cell proliferation. CCK-8 assay results to show the effects of different groups on viability of (**A**) Raji cell, (**B**) BJAB cell, and (**C**) Pfeiffer cell. Data shown are means ± SD from three separate experiments (n = 3).

The cell viability of Raji, BJAB, and Pfeiffer cells was evaluated using CCK-8 method at 24 h and 48 h, respectively. Based on the IC50 results, we calculated the synergistic index of the combination in each cell line. The results are shown in Fig. 5 and Table 2. Although the IC50 values of different cells in different experimental groups were different, they all showed that the IC50 value decreased with the prolongation of treatment time. In all 3 cell lines that were treated with the same time, the IC50 value of DOX was higher than L-DOX, which was higher than L-DOX + CUR. At each time point, the CI was less than 1 at the respective IC50 concentrations. These suggested the degree of synergies in combination therapy, except that the combination of L-DOX and L-CUR at 24 h showed antagonistic effect in Raji cells.

**Table 2:** The IC_50_ (μM) and CI of different groups on Raji, BJAB, and Pfeiffer cell at 24 h and 48 h (±SD).

Raji	Pure drug			Loaded drug		
	CUR	DOX + CUR	CI	L-CUR	L-DOX + CUR	CI
24 h	9.47 ± 0.85	3.15 ± 0.350	0.967	7.39 ± 0.600	2.69 ± 0.304	1.03
48 h	6.83 ± 0.600	0.02 ± 0.002	0.475	0.37 ± 0.035	0.02 ± 0.002	0.810
**BJAB**	**Pure drug**			**Loaded drug**		
	**CUR**	**DOX + CUR**	**CI**	**L-CUR**	**L-DOX + CUR**	**CI**
24 h	6.91 ± 0.600	2.35 ± 0.202	0.884	6.46 ± 0.625	2.63 ± 0.250	0.968
48 h	6.32 ± 0.600	0.02 ± 0.002	0.094	2.01 ± 0.200	0.02 ± 0.002	0.721
**Pfeiffer**	**Pure drug**			**Loaded drug**		
	**CUR**	**DOX + CUR**	**CI**	**L-CUR**	**L-DOX + CUR**	**CI**
24 h	7.98 ± 0.851	2.48 ± 0.200	0.998	10.0 ± 0.578	0.79 ± 0.133	0.565
48 h	6.11 ± 0.600	0.74 ± 0.075	0.605	3.14 ± 0.301	0.23 ± 0.020	0.758

### Apoptosis analysis

**Figure 6:**
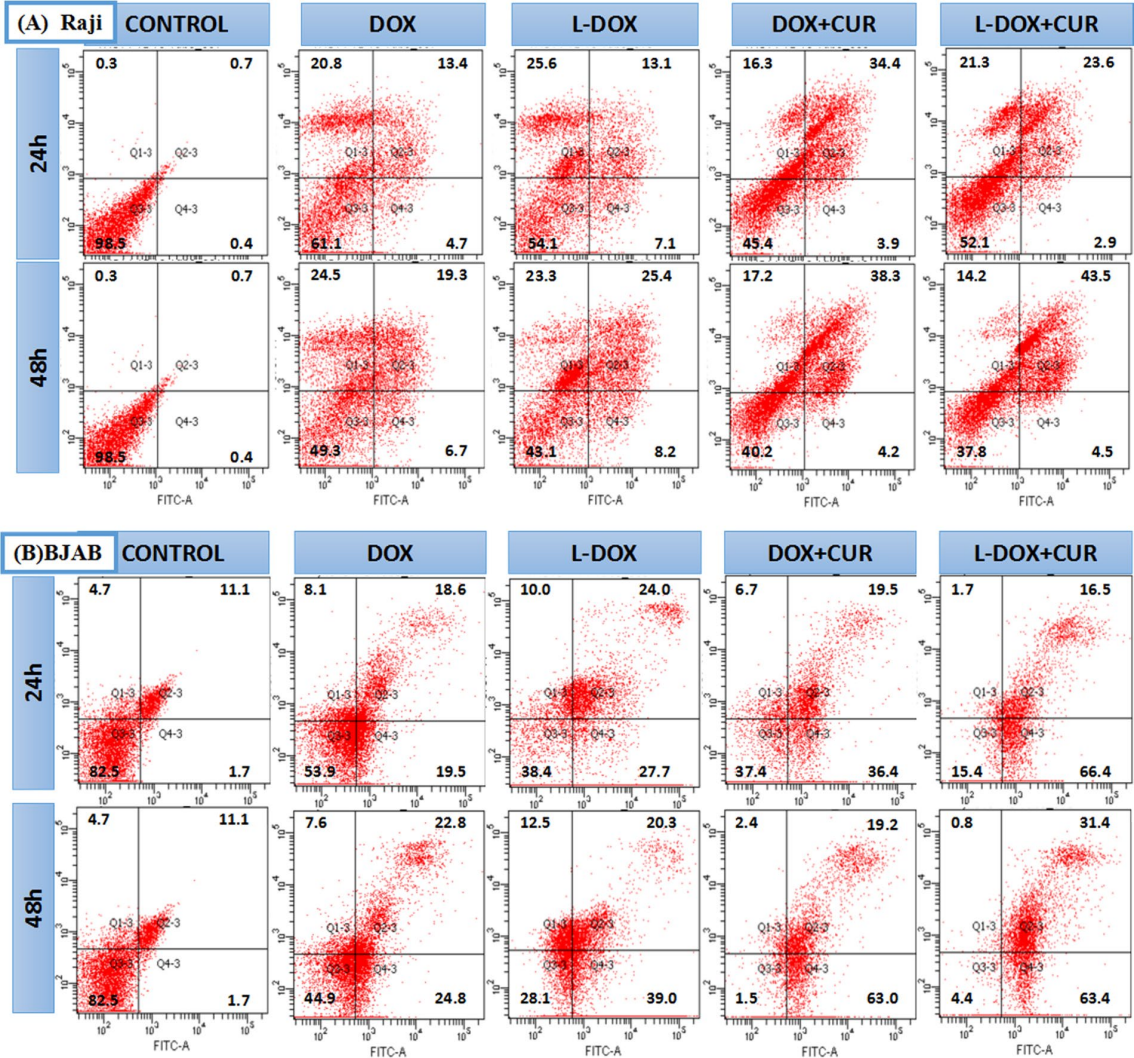
Analysis of apoptosis. (**A**) Flow cytometry results of the apoptosic rates (%) on (**A**) Raji cell and (**B**) BJAB cell, which was induced by DOX or L-DOX or DOX + CUR or L-DOX + CUR for 24 h and 48 h, all groups of DOX were 0.5 μM, and Annexin V-FITC/PI Apoptosis Detection Kit was performed.

To further explore the mechanism of action of the study drugs, we examined the effects DOX, L-DOX, DOX + CUR and L-DOX + CUR (DOX 0.5 μM) in Raji and BJAB cells using Annexin V-FITC/PI double staining for 24 h and 48 h. The flow cytometry results are shown in Fig. 6. The results are in 4 quadrants. The upper left (PI-FITC+) indicates dead cells, the upper right (PI + FITC+) indicates late apoptotic cells, and the lower left (PI-FITC−) indicates normal living cells. The lower right (PI-FITC+) suggests early apoptotic cells. The proportion of apoptosis is calculated as the ratio of the upper right and lower right cells to the total number of cells. The apoptosis rate of each of the two cell groups was higher than that of the control group, P < 0.001. The results of the pairwise comparison is shown in Table 3. Apoptosis rate of Raji cells was higher, in DOX + CUR group than the DOX group (24 h P = 0.002, 48 h P = 0.013). Apoptosis rate of Raji cells in L-DOX + CUR group was higher than the L-DOX group (P = 0.044). The apoptosis rate of BJAB cells was consistent at 24 h and 48 h. The apoptosis rate of BJAB cells was higher in L-DOX + CUR than DOX + CUR group, which is higher than DOX group (both in pairs and P < 0.05). The apoptosis rate of BJAB cells was also higher in L-DOX + CUR group that that of L-DOX group (P < 0.001). The results showed that L-DOX + CUR induced the apoptosis of Raji cells and BJAB cells more strongly than L-DOX, and L-DOX was not inferior to DOX, indicating that one of the synergistic mechanisms of DOX and CUR is to promote apoptosis. Furthermore, this effect is the most significant in BJAB cells.

**Table 3:** Apoptotic rate of BJAB and Raji cells (%) at 24 h and 48 h.

Cell	Time	Groups					*P*
		Control	DOX	L-DOX	DOX + CUR	L-DOX + CUR	
BJAB	24 h	12.8	38.1	51.7	55.9	82.9	*, Δ, δ, φ
	48 h	12.8	57.6	59.8	82.2	94.8	**,δ, φ
Raji	24 h	1.1	18.1	20.2	38.3	26.5	**
	48 h	1.1	26.0	33.6	42.5	48.0	Δ, ξ

Note: After chi-square test, pairwise comparison, *indicates that the apoptotic rate is significantly higher in the L-DOX group than in the DOX group (P < 0.05), **indicates that the apoptotic rate in the DOX + CUR group is significantly higher than that in the DOX group (P < 0.01). Δ indicates that the apoptotic rate is significantly higher in the DOX + CUR group than in the DOX group (P < 0.05), δ indicates that the apoptotic rate is significantly higher in the L-DOX + CUR group than in the DOX + CUR group (P < 0.01), and ξ indicates apoptosis. The rate of L-DOX + CUR group was significantly higher than that of L-DOX group (P < 0.05). φ indicates that the apoptotic rate was significantly higher in the L-DOX + CUR group than in the L-DOX group (P < 0.01).

**Figure 7:**
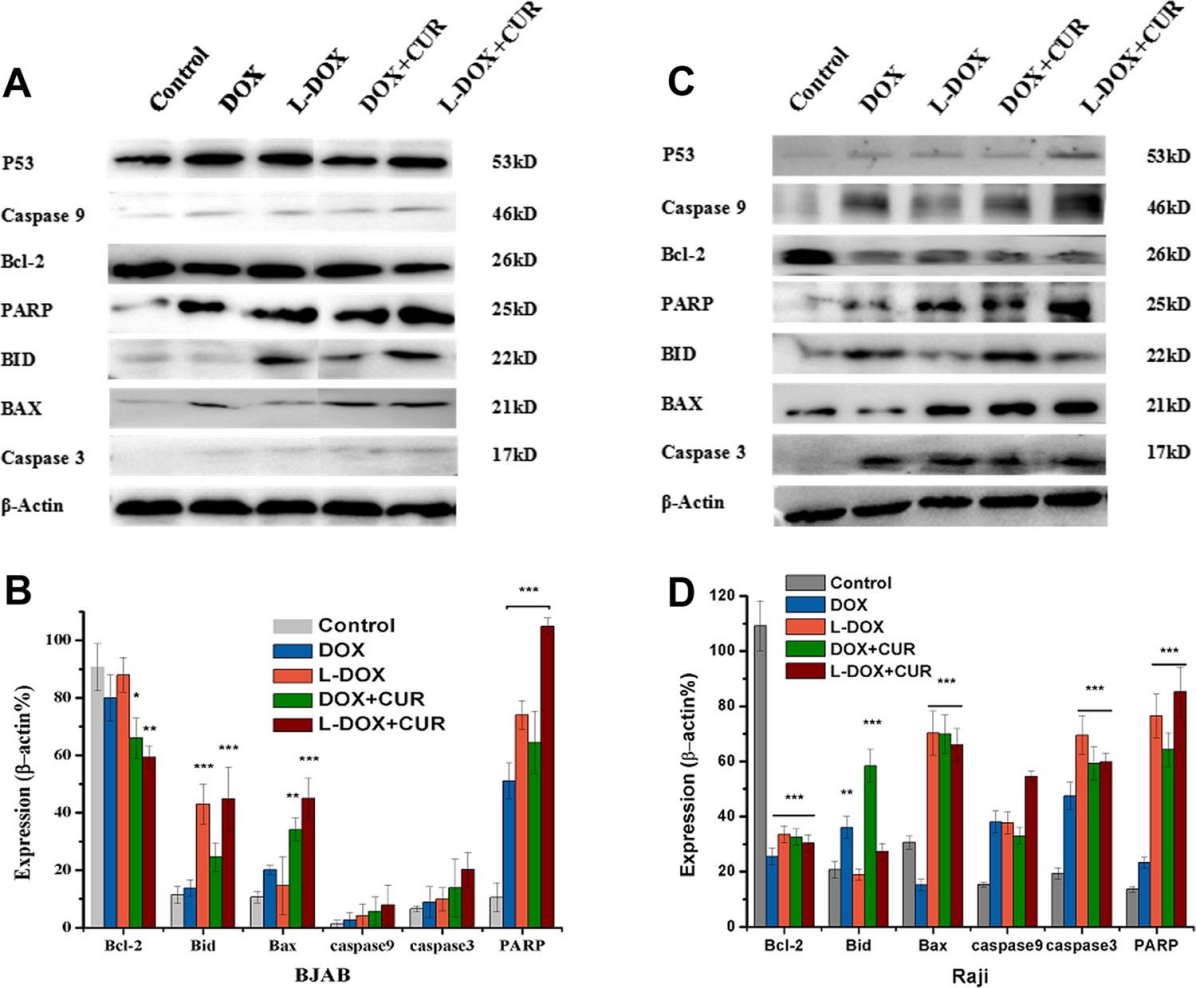
Analysis of apoptotic pathway proteins. Western Blot result of apoptotic protein expression on (**A**,**B**) BJAB cell and (**C**,**D**) Raji cell after incubated with DOX or L-DOX or L-DOX + CUR for 24 h (DOX 0.5 μM).

To further analyze the mechanism of enhanced apoptosis seen in the nano drug-loading system, we again treated BJAB and Raji cells with the same dose (DOX 0.5 μM) of DOX, L-DOX, DOX + CUR and L-DOX + CUR. Flow cytometry analysis showed that this concentration was sufficient to promote apoptosis after 24 hours, and the expression of apoptotic proteins in each drug group for 24 h was evaluated using the Western Blot. The results and analysis are shown in Fig. 7. In BJAB cells and Raji cells, the expression of P53 was slightly increased compared with the control, but it was not statistically significant. The L-DOX and L-DOX + CUR groups showed a significant increase in PARP levels (P < 0.001). in L-DOX + CUR group, the expression of Bcl was decreased and the expression of Bax was increased compared to DOX + CUR group (P < 0.05). However, the difference between the two lymphoma cells was also found. BJAB cells showed significant increase in PARP in each treatment group (P < 0.001). Comparison between groups PARP expression in L-DOX was higher than DOX, and L-DOX + CUR is higher than L-DOX in BJAB cells. In contrast, Raji cells showed a decrease in Bcl-2 (P < 0.001), and there was an increase of Bax (P < 0.001) and caspase-3 (P < 0.001) except the DOX group. The pro-apopotic protein Bid, which is involved in the external pathway of apoptosis, was significantly increased in the nano-drug groups in BJAB cells (P < 0.001), while in the Raji cells Bid was significantly increased in the pure drug group (P < 0.01). These results suggested that in both BJAB and Raji lymphoma cells, DOX-based drugs can induce apoptosis and the nano-carrier system can enhance this pro-apoptotic ability and may also involve other apoptosis pathways outside the Bcl-2 signaling.

### Maximum tolerated dose (MTD) study

**Figure 8:**
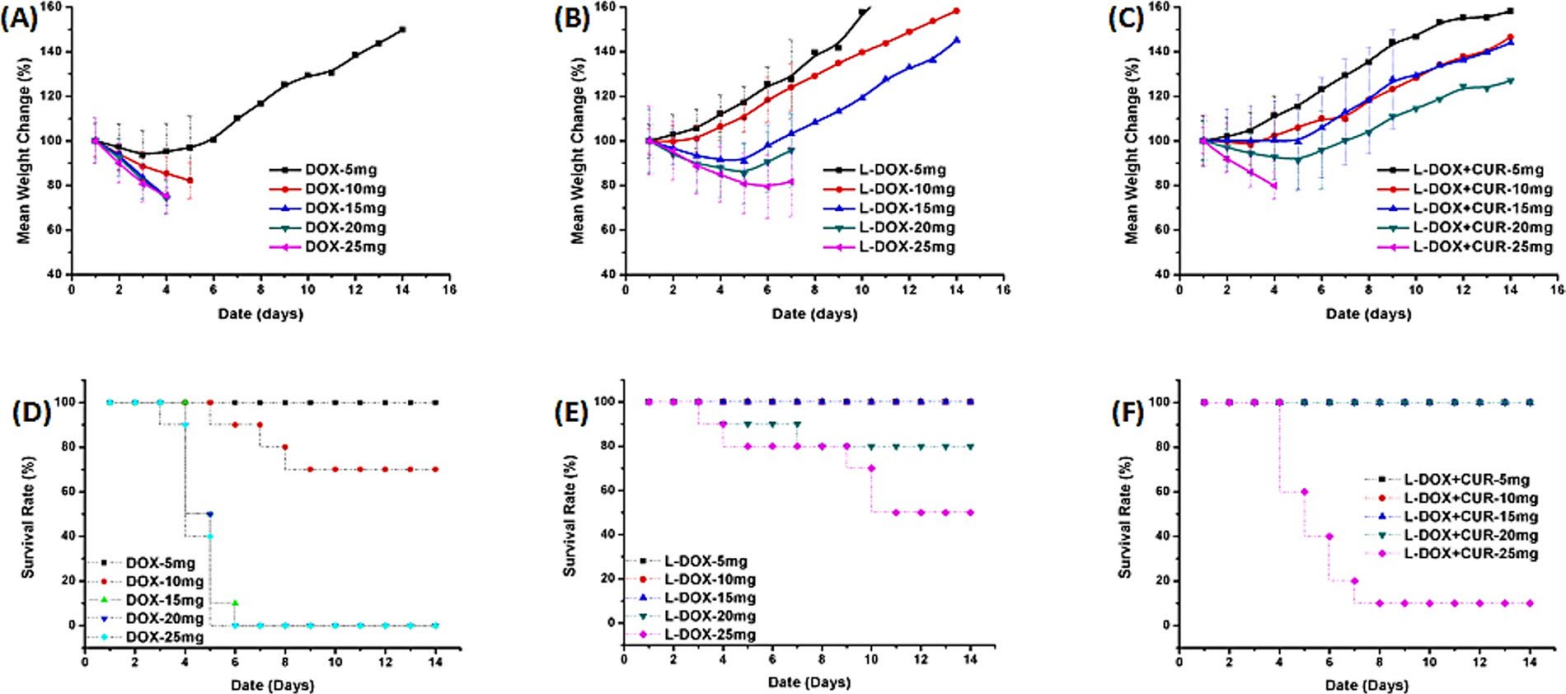
Maximum Tolerated Dose (MTD) Study. MTD on Kunming mice treated with DOX or L-DOX or L-DOX + CUR. (**A**–**C**) mean weight change rate; (**D**–**F**) survival rate.

To initially explore the safety of our nanomedical drug system, Kunming mice were used for MTD testing. The DOX group, L-DOX group and L-DOX + CUR group were examined, and the doses were all normalized in DOX, including 5 mg/kg, 10 mg/kg, 15 mg/kg, 20 mg/kg, and 25 mg/kg. The results are shown in Fig. 8 show that mouse death occurred in all concentrations of DOX except 5 mg, L-DOX 20 mg and L-DOX + CUR 25 mg. The mice with weight loss of more than 20% died in the short term, and there were gastrointestinal toxic reactions such as anorexia and diarrhea before death. Hence, the MTD of DOX in Kunming mice was 5 mg/kg, L-DOX was 15 mg/kg, and L-DOX + CUR was 20 mg/kg, which was the highest, respectively. These results suggested that mPEG-b-P (Glu-co-Phe) enhanced DOX tolerance after carrying DOX, and the tolerance was further improved after co-loading CUR, indicating the increase of the therapeutic window. Based on these results, we selected the single drug injection dose of DOX 3 mg/kg for in vivo efficacy studies.

### *In vivo* anti-lymphoma effect study

**Figure 9:**
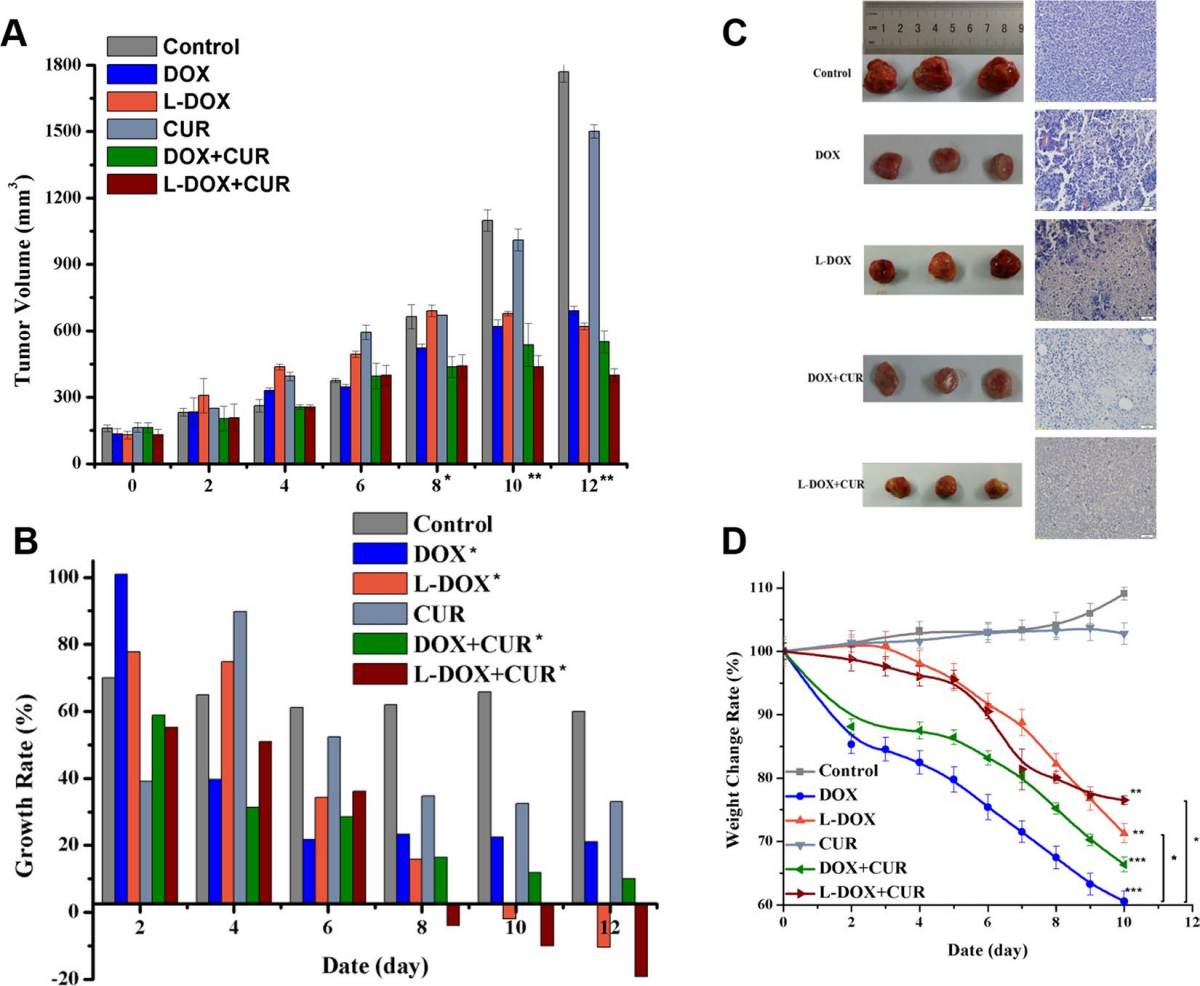
*In vivo* efficacy studies. (**A**) Change of tumor volume demonstrates the inhibition of BJAB bearing SCID mice xenograft. (DOX 9 mg/kg). (**B**) Growth rate of tumor volume demonstrated the inhibition of BJAB bearing SCID mice xenograft. (DOX 9 mg/kg). (**C**) Gross view and HE staining results of the tumors. (**D**) weight change rate of BJAB bearing SCID mice during treatment. (**A,B**) One-way ANOVA test, between groups, *indicates P < 0.05, **indicates P < 0.001. (**D**) Due to the death on the 12th day, the mice died, so the weight change analysis, the data was weighed until the 10th day. After one-way analysis of variance, each treatment group and the control group were compared. **Indicates P < 0.01, ***indicates P < 0.001 when compared with the treatment group. *Indicates P < 0.05. When the DOX group was compared with the L-DOX group, P = 0.013, and the DOX group also decreased compared with the L-DOX + CUR group, P = 0.016.

In order to evaluate the in vivo effect of the nano-drug system on lymphoma, we established BJAB cell-bearing SCID mice as a mouse model of xenograft. The tumor volume measurement results are shown in Fig. 9A. Comparing the treatment groups, the tumor volume began to show statistical difference on the 8th day (p = 0.031), and on the 10th and the 12th days (P = 0.000). Although the shrinkage of the tumor has not yet occurred until the end of the treatment observation, the growth rate of the tumor has decreased as shown in Fig. 9B. Specifically, compared with the control group, the CUR group (not shown), DOX Group, L-DOX group, DOX + CUR group, and L-DOX + CUR group, P values were 0.235, 0.021, 0.003, 0.003, 0.001, respectively. Moreover, both L-DOX and L-DOX + CUR groups showed negative tumor growth, and the L-DOX + CUR group was the earliest to exhibit negative growth.

At the same time, we also weighed the mice and observed the toxicity of each treatment group. The results are shown in Fig. 9D. Except that the weight of the control and CUR groups that did not decrease (P = 0.753), the other groups showed different degrees of weight loss. Compared with the control group, the DOX group, L-DOX group, DOX + CUR group and L-DOX + CUR group showed significant decrease in body weight (P value <0.001, 0.010, <0.001, 0.008 respectively). The most significant decline was in the DOX group, followed by the DOX + CUR group, which exceeded 30% of the basal body weight (DOX group appeared on day 8, DOX + CUR group appeared on day 10), and the least decrease was The L-DOX + CUR group, up to day 10, was 23%, and the rate of decline was slower.

### Pathological analysis

In order to further clarify the effect of the treatment group on the tumor tissue, the sections of tumor, heart, liver, spleen, lung, kidney and bone marrow were further observed by HE staining. Different degrees of tumor necrosis-like changes were observed in the treatment group (necrotic cells were pale due to inability to be stained by hematoxylin/eosin), and the results are shown I Fig. 9C. The degree of necrosis of the tumor in the L-DOX + CUR group is the heaviest. There were no obvious abnormal changes (not shown) in the HE slices of the main organs in each group.

## Discussion

We used mPEG-b-P (Glu-co-Phe) as a carrier to carry DOX by electrostatic interaction to synthesize nano-doped doxorubicin (L-DOX) with a drug loading of 10.6%. It has been confirmed by *in vitro* experiments that L-DOX has a stronger ability to enter human invasive B-cell lymphoma cells (Raji, BJAB, and Pfeiffer cells) than DOX, and to release DOX after entering cells, and has a stronger cytotoxicity. These results provided a basis for further studies of nanoparticles that are co-loaded with DOX and DUR.

Endocytosis, also known as inoculation, is the process of transporting extracellular substances into cells through the deformation movement of the plasma membrane. Endocytosis can be divided into three types depending on the size of the invading material and the mechanism of entry into the cell: phagocytosis, swallowing, and receptor-mediated endocytosis. The nanomaterials we studied have a particle size of about 130–150 nm, so they depend on swallowing to enter the cell, also known as endocytosis. It is a non-selective endocytosis process that continuously takes up nanoparticles in the extracellular matrix. The material that is swallowed is usually a liquid or a lysate, and the small vesicles formed have a diameter of less than 150 nm. DOX has autofluorescence. DOX can enter cells quickly through passive diffusion. L-DOX is a macromolecule that enters cells through endocytosis. After fully washing away the extracellular fluid, confocal microscopy was applied. The intensity of DOX fluorescence signal was observed to study the release of L-DOX after entering cells. However, many previous studies on the concentration of DOX in cells suggested that DOX has the strongest intake after 1 hour with a treatment of 5.0 μM, and prolonged with time for more than 4 hours. Hence, in this study we selected two time points, 1 h and 3 h.

In tumor tissue, the extracellular pH is about 6.5~7. In the normal tissue, the extracellular fluid pH is 7.4, the intracellular endosomal pH is 5.0~6.5, and the lysosome pH is 4.5~5.0. The release ratio of L-DOX prepared in the present study began to increase significantly under a pH less than 6.8. Although we only examined the level of pH up to 5.5, according to the curve, further reduction in pH may still achieve higher release. This pH level is well within the pH range of endosomes and lysosomes, allowing L-DOX to achieve relative selectivity for intracellular release, and if released in vivo presumably reduce the loss of L-DOX in normal tissues during blood circulation. Once L-DOX enters the tumor cells, it is released quickly, thereby exerting a better anti-tumor effect and relatively reducing the side effects^19^. The results of confocal microscopy confirmed the enrichment of DOX in the nucleus that are consistent with the primary anti-tumor mechanism of DOX, which inserts the DNA double helix structure, covalently binds to the essential protein for DNA replication, prevents transcription, and leads to cell death^20–23^. The fluorescence signal site of L-DOX is consistent with DOX, indicating that L-DOX acts by releasing DOX after endocytosis into the cell; and this signal is higher than the DOX of the same action time, suggesting that the endocytosis efficiency of L-DOX is higher than DOX. Furthermore, we found significant growth inhibition over time, and apoptosis-like changes, which is consistent with DOX-induced apoptosis mechanism^24^. Our CCK-8 study confirmed that L-DOX has a certain therapeutic advantage over DOX, a result was similar to previous studies^25^.

To explore the effects of the L-DOX-CUR system on lymphoma cells, we first observed directly by confocal microscopy and found that the CUR signal could not be detected in each cell at 1 h. This was related to the time dependence of entry of CUR into the cells. The time for our drug to treat cells was much shorter than that reported in the literature (e.g., 12~24 h)^14,26^. At the same time, Raji cells grew in a pellet, and the diameter of Pfeiffer cells was small. The differences between groups in endocytosis were observed. There were no significant differences among the three cells. Therefore, the final concentration was equivalent to DOX 5 μM, and the DOX:CUR molar ratio was 1:1.23. It was found that CUR and DOX were different in the intracellular enrichment site, and the intracellular concentration was increased after the combination of the two drugs, and the effect of the nano-carrier L-DOX-CUR was stronger.

The mechanism of interaction between CUR and cell membrane is still unclear. CUR is a small molecule that is insoluble in water. Its ability to penetrate into cells is limited, and cell membrane is its main barrier. With the help of nanocarriers, CUR is more water soluble and can be endocytosed. The nanocarrier enhances the ability of CUR to enter cells. Furthermore, we observed that CUR was mainly concentrated in the area outside the nucleus, unlike DOX, which was mainly concentrated in the nucleus, suggesting that the mechanism of the two at the molar ratio of 1:1.23 is different. Further detection of intracellular DOX concentration by flow cytometry showed that the L-DOX + CUR group was the highest, higher than the DOX + CUR group and higher than the L-DOX group, but the DOX + CUR group was higher than the DOX group. Obviously, there may be a synergistic effect between the two-drug combination, and this effect is more pronounced in the nanocarrier system.

Subsequently, the toxic effects of the drug on the cells were detected by CCK-8 method. We found that DOX and DUR showed synergistic killing effect, and the degree of synergy was different in the three kinds of cells, and BJAB cells showed strong synergistic effect (CI < 0.4). Our results are consistent with previous studies^27–29^. By calculating and comparing the IC50 values, it was also confirmed that the effect time of each drug group was prolonged, and the killing effect was enhanced, especially CUR, which showed cytotoxicity at almost 48 hours. Reports of various CUR effects show that large doses are often required to exert a killing effect. The threshold of this dose varies from cell to cell in similar reports^14,26,30^. However, concentrations used in this study (~12.3 μM), do not kill and inhibit proliferation lymphoma cells. In the cytotoxicity test, the morphology of the cells was observed under the microscope, and the cells were found to have “apoptosis-like changes”. The literature also reported that one of the important mechanisms of DOX is the induction of apoptosis. Our results indicated that the L-DOX + CUR induced the apoptosis of in cells stronger than L-DOX, and L-DOX was not inferior to DOX.

Western Blot results showed the same and different effects of the study drug system on the apoptosis mechanism in Raji and BJAB cells. Firstly, the DOX-based drugs showed a decrease in Bcl-2 protein and increased expression of proteins in the downstream apoptotic pathway. These results confirmed the involvement of mitochondrial apoptosis pathway induced by DNA damage in the Bcl-2 family. The anti-apoptotic protein represented by Bcl-2 and the pro-apoptotic protein represented by Bax affect the release of cytochrome c (CytC) on the mitochondrial membrane, initiate the caspase3 apoptotic pathway signaling, and promote the apoptosis when the ratio of Bax to Bcl-2 is high^7,20,23,31–34^. Moreover, the L-DOX + CUR group showed a stronger induction of apoptosis than the other groups. In addition, PARP is a genomic monitoring and DNA repair enzyme. When a large amount of DNA damage is not effectively repaired, the damaged cells start to undergo apoptosis. We found the product of PARP cleavage (25KD), the appearance of which means that PARP loses its enzyme activity, which is an important indicator of apoptosis, and is also an indicator of caspase3 activation, as well as cell instability. For further comparative analysis between the two cell groups, we found ct was higher in L-DOX than DOX, and L-DOX + CUR was higher than DOX + CUR (74.0% vs. 51.2%, P = 0.018) in BJAB cells. These indicate nano-drug delivery system promotes the apoptotic effect by increasing the amount of effective drug that enters the cell. Additionally, L-DOX + CUR group exhibits higher apoptosis L-than DOX group in BJAB cells, suggesting that this enhancement also benefits from the involvement of CUR^16,20,35,36^. In Raji cells, the treatment group showed an increase in the ratio of Bax/Bcl-2, but there was no significant difference in the PARP between the groups except the DOX group, and the expression of Bid in the nano drug-loaded group increased significantly, suggesting that other apoptotic pathways may also be involved in the apoptosis of Raji cells. Therefore, it is certain that the L-DOX + CUR system can achieve therapeutic goals by enhancing the apoptotic pathway through the mitochondrial apoptotic pathway for the invasive B-cell lymphoma represented by BJAB and Raji cells.

In the pre-experiment, Raji cell-bearing SCID mice showed low surface tumor formation rate, strong invasiveness, rapid tumor formation, and bone marrow involvement, which was consistent with the characteristics of Butkitt lymphoma disease. However, our results were not consistent with some reports that showed the success using subcutaneous injections of 10^6^~10^7^ cell via the tail vein. This may be related to the source of the cells and the type of tumor-bearing mice. In this study, BJAB cells were used for the first time. Under the subcutaneous implantation method, the tumor-bearing SCID mice had a tumor formation rate of more than 80%, a short tumor formation time (8–12 days), and the tumor viability and body weight after tumor formation. There is almost no decline, and the tumor can be maintained for about 1 week in a certain volume range. It is an ideal model for studying large B-cell lymphoma.

Based on the results of MTD experiments in Kunming mice, L-DOX increased the tolerance of DOX to 3-fold and L-DOX + CUR to 4-fold, confirming that mPEG-b-P (Glu-co-Phe) drug carrier system reduced the toxicity of DOX and increased the therapeutic window. Further, the anti-tumor experiment was carried out on the constructed BJAB cell-bearing SCID mouse model. The results showed that the L-DOX + CUR group had the strongest anti-tumor effect, and the mice in the therapeutically effective group had the least weight loss. Although the tumor did not shrink due to treatment, there was a negative volume growth in the L-DOX and L-DOX + CUR groups, suggesting that if the observation time is prolonged, the tumor is expected to gradually shrink in L-DOX + CUR group. Also, it can be seen from the observation of tissue section microscopy that the degree of necrosis was the most in the L-DOX + CUR group. At the same time, CUR monotherapy did not show significant anti-tumor effect, but the effect was enhanced after combined with DOX, which was consistent with *in vitro* cytotoxicity studies with DOX, suggesting there is a synergistic effect.

## Conclusion

The polymer nanomaterial mPEG-b-P (Glu-co-Phe) was used as a carrier to carry doxorubicin (L-DOX). Compared with traditional DOX, L-DOX has stronger invasive B-cell lymphoma cells. Raji, BJAB, Pfeiffer). The mPEG-b-P (Glu-co-Phe) can be used to generate the L-DOX + CUR system to further enhance the ability of DOX to enter these cells. At the molar ratio of 1:1.23, there is a synergistic effect between DOX and CUR in pure drug and nano drug-loading system, and this effect is the strongest in BJAB cells. L-DOX + CUR is more toxic to each cell than L-DOX and stronger than DOX. L-DOX + CUR enhances DOX-induced apoptosis in the endogenous mitochondrial pathway and may involve other apoptotic mechanisms. L-DOX + CUR can increase the MTD of DOX in Kunming mice by more than 4 times, significantly reducing its toxicity. Preparation of tumor-bearing SCID mice by subcutaneous implantation of 5 × 10^6^ BJAB cells is a feasible method to establish the mouse model of large B-cell lymphoma in vivo. L-DOX + CUR treatment of BJAB cell-bearing SCID mice was more effective and less toxic than L-DOX or DOX. In summary, we demonstrated that the high molecular weight mPEG-b-P (Glu-co-Phe) co-loaded with doxorubicin and curcumin has high anti-lymphoma effect and low toxicity. Our results support the application of polymeric nanomaterials to carry traditional chemotherapeutic drugs and provide a theoretical basis for the treatment of lymphoma with synergistic drugs. The developed novel nano-drug delivery system has considerable clinical value.
